# Laser Interstitial Thermal Therapy in Grade 2/3 *IDH1/2* Mutant Gliomas: A Preliminary Report and Literature Review

**DOI:** 10.3390/curroncol29040209

**Published:** 2022-04-08

**Authors:** Gabrielle W. Johnson, Rowland H. Han, Matthew D. Smyth, Eric C. Leuthardt, Albert H. Kim

**Affiliations:** 1 Department of Neurosurgery, Washington University School of Medicine, St. Louis, MO 63110, USA; gabrielle.johnson@wustl.edu (G.W.J.); rowland.han@wustl.edu (R.H.H.); leuthardte@wustl.edu (E.C.L.); 2 Department of Neurosurgery, Johns Hopkins All Children’s Hospital, St. Petersburg, FL 33701, USA; msmyth4@jhmi.edu; 3 Brain Tumor Center, Siteman Cancer Center, Washington University School of Medicine, St. Louis, MO 63110, USA

**Keywords:** glioma, *IDH1* mutation, *IDH2* mutation, laser interstitial thermal therapy, astrocytoma, oligodendroglioma

## Abstract

Laser interstitial thermal therapy (LITT) has become an increasingly utilized alternative to surgical resection for the treatment of glioma in patients. However, treatment outcomes in isocitrate dehydrogenase 1 and 2 (*IDH1/2*) mutant glioma, specifically, have not been reported. The objective of this study was to characterize a single institution’s cohort of *IDH1/2* mutant grade 2/3 glioma patients treated with LITT. We collected data on patient presentation, radiographic features, tumor molecular profile, complications, and outcomes. We calculated progression-free survival (PFS) and tested factors for significant association with longer PFS. Overall, 22.7% of our cohort experienced progression at a median follow up of 1.8 years. The three- and five-year estimates of PFS were 72.5% and 54.4%, respectively. This is the first study to characterize outcomes in patients with *IDH1/2* mutant glioma after LITT. Our results suggest that LITT is an effective treatment option for *IDH1/2* mutant glioma.

## 1. Introduction

Since its first description in 1983 [1] and application in the treatment of brain lesions in 1990 [2], indications for laser interstitial thermal therapy (LITT) within the central nervous system (CNS) have continued to expand. While initially used in the treatment of recurrent glioblastoma (GBM), LITT has become more common in the treatment of primary CNS neoplasms [3,4,5,6], brain metastases [7,8], radiation necrosis [7,9,10], and epilepsy [11,12]. Retrospective studies have shown that LITT is a reasonable and at times favorable alternative to standard of care surgical resection in gliomas, particularly in cases where lesions are deep-seated or if the patient is not a good candidate for open surgical resection [4,7,13,14]. 

Gliomas with mutations in *isocitrate dehydrogenase* (*IDH*) *1* and *2*, first discovered in genomic analysis of GBM, are a molecularly distinct subtype of diffuse glioma associated with younger age of diagnosis and longer overall survival compared to *IDH1/2* wild-type gliomas [15,16,17]. While many studies of glioma have historically combined *IDH1/2* mutant and wild-type cohorts when analyzing treatment outcomes in glioma patients, recently, more concerted efforts have been made to analyze the outcomes of *IDH1/2* mutant gliomas as a distinct entity [18,19,20]. Currently, the standard of care in the treatment of *IDH1/2* mutant gliomas is maximal, safe surgical resection, with adjuvant radiotherapy and specific chemotherapy dependent on molecular subtype and grade [21]. Repeat surgery, particularly in the case of recurrent high-grade glioma, is also commonplace [22,23].

Over the last decade, LITT has become an increasingly utilized modality in the treatment of gliomas. While LITT has been shown to be an effective, well-tolerated alternative to open surgical resection in both low- [4,24,25] and high-grade [14,26,27] gliomas, LITT treatment outcomes specific to *IDH1/2* mutant grade 2 and 3 gliomas have not been studied. The objective of the present study was to describe the clinical and histopathological presentation, treatment, and outcomes of patients with *IDH1/2* mutant grade 2/3 gliomas treated with LITT at a single institution, to determine the progression-free survival of these patients, and to compare their clinical outcomes with previously published findings. 

## 2. Materials and Methods

**Figure 1:**
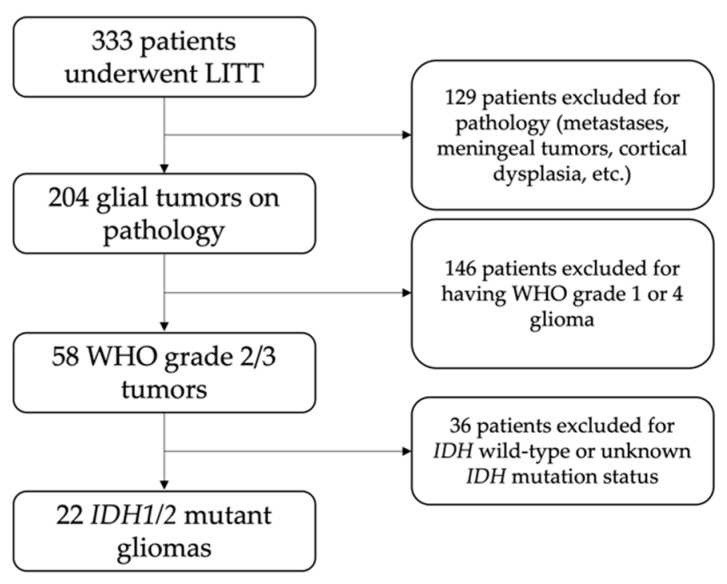
Flowchart of patient selection.

The study was conducted with the approval of the institutional review board (IRB ID# 201409046, approval date: 3 February 2022). This study was a single-institution, retrospective case series of patients treated at Washington University, Barnes Jewish Hospital, and St. Louis Children’s Hospital with LITT for *IDH1/2* mutant grade 2 or 3 gliomas from 2014 to 2021. Patients who underwent LITT at our institution were identified via combination of case log databases and departmental billing records. Patients were excluded if their pathology was not consistent with a glial neoplasm, was determined to be WHO grades I or IV, was *IDH1/2* wild-type, or was of an unknown *IDH1/2* mutation status (Figure 1). 

Data extracted from the electronic medical record included patient demographics, clinical presentation, radiographic characteristics, treatment prior to LITT, LITT operative details, adjuvant treatment, and outcomes after treatment to better classify the presentation and outcomes of these patients. Tumor volume was calculated using the formula (tumor volume) = (antero-posterior diameter) × (transverse diameter) × (craniocaudal diameter)/2. In cases where a patient presented with more than one lesion, tumor volumes were combined for analysis.

We collected tumor genetic information to classify gliomas by molecular subtype according to 2021 WHO guidelines [28]. *IDH1/2* mutation status was determined by immunohistochemistry (R132H antibody) or DNA sequencing. Chromosome 1p and 19q deletion status was determined using fluorescence in situ hybridization. Using this information, we reclassified all tumors into the following categories: (1) oligodendroglioma (ODG; *IDH1/2* mutant with 1p/19q co-deletion) and (2) diffuse astrocytoma (DA; *IDH1/2* mutant without 1p/19q co-deletion or with *TP53* and *ATRX* mutations). Patients with non-glial neoplasms, no *IDH1/2* mutation on immunohistochemistry or sequencing, or inconclusive *IDH1/2* status were excluded from this study.

Our primary outcome of interest in this study was progression-free survival (PFS). Progression was defined as an increase in area of enhancement of a previously treated enhancing lesion and/or new or increasing mass lesions on MRI. PFS was measured from the date of initial LITT to the date of documented radiographic progression. Secondary outcomes of interest included complications, operative details, and need for repeat LITT. In our analysis, we considered pre-planned, staged LITT treatments as one treatment. 

A literature search was performed to identify studies analyzing treatment outcomes and progression-free survival of *IDH*-mutant cohorts who underwent craniotomy or LITT. Studies analyzing *IDH*-mutant grade 2 or 3 glioma cohorts that reported survival outcomes were reviewed. 

Statistical analyses were performed using SPSS version 28 (IBM Corporation, Armonk, NY, USA). Categorical variables were reported as percentage of the total cohort. Continuous variables were evaluated for normality; normally distributed continuous variables were reported with mean and standard deviation, and non-normally distributed continuous variables were reported with median and range. 

Median time to progression was obtained using Kaplan–Meier (KM) product-limit estimates of the survival function and were graphically represented using KM curves. If the survival estimate was above 50% at the end of observation time, a “not reached” indicator was used. KM survival tables were used to estimate PFS at 3 and 5 years. KM curves were constructed for the overall cohort, first line versus salvage treatment, prior extent of resection, extent of ablation, and tumor pathology. Log-rank testing was conducted to identify factors independently associated with differences in PFS. Univariate logistic regression was used to identify factors independently associated with progression. Factors with *p* < 0.10 were retained for multivariable analysis (Cox proportional hazard testing and multivariable logistic regression). 

Patient consent was not required due to the retrospective nature of the study.

## 3. Results

### 3.1. Baseline Characteristics and Presentation

Twenty-two patients who underwent LITT for *IDH1/2* mutant grade 2/3 glioma at a mean age of 46.6 years were included in this study. Their demographics, presenting symptoms, and pre-LITT treatment details can be found in Table 1. Of these patients, 63.6% were male, 86.4% were white, and 95.5% were alive at the time of chart review. The series included one death due to an unknown cause but thought not to be tumor-related (Case #21). Sixteen patients (72.7%) were symptomatic at presentation, with seizure (40.9%) and headache (22.7%) as the most common symptoms. The median (range) pre-LITT Karnofsky Performance Score (KPS) was 85 (60–100). The median (range) initial tumor volume was 5.6 (0.18–48.38) cm^3^. 

Treatment prior to LITT was common, with 72.7% of patients having undergone pre-LITT treatment. Surgical resection was the most common pre-LITT treatment (14 patients, 63.6%), with 57.1% of these patients undergoing gross total resection (GTR) and 42.9% undergoing subtotal resection (STR). Ten patients (45.5%) underwent chemotherapy, with most patients (90%) undergoing chemotherapy with temozolomide. Radiation therapy was also common, with 12 (54.4%) of patients undergoing treatment. Biopsy was less common (four patients, 18.2%), and radiosurgery was the least common pre-LITT treatment (one patient).

### 3.2. Tumor Pathology

*IDH1/2* mutant gliomas were re-classified according to the 2021 WHO guidelines into oligodendroglioma (ODG, *IDH1/2* mutant and 1p/19q co-deleted) and diffuse astrocytoma (DA, *IDH1/2* mutant without 1p/19q co-deletion). Of the 22 patients in the study, 12 patients (54.5%) had ODG and 10 (45.5%) had DA. Most tumors (59.1%) were grade 2, while the remaining nine (40.9%) were grade 3. Only one patient (Case #2) had a mutation in *IDH2* (R127K); the remaining patients had mutations in *IDH1*. The most common *IDH1* mutation was R132H (90.1%), followed by R132C (4.5%). Other mutations in each patient’s tumor, along with Ki-67 proliferative index information, can be found in Table 2. 

### 3.3. LITT Treatment

LITT operative details and outcomes are detailed for each case in Table 3. LITT was the first-line treatment in 36.4% of our cohort. The median (range) volume ablated in this cohort overall was 95% (65–100%). The mean ± standard deviation (SD) operative time was 211.2 ± 77.7 min, and the mean anesthesia time was 342.5 ± 80.3 min. Overall, three patients in our cohort (13.6%) experienced perioperative complications after LITT. Two patients (9%) in the cohort suffered from perioperative complications after their initial LITT procedure. Of these, one patient (Case #12) developed seizures in the post-operative period, while the other (Case #19) was treated for deep vein thrombosis. A third patient (Case #1) suffered from perioperative complications after a second, non-staged LITT procedure for residual tumor. Her course was complicated by severe cerebral edema requiring decompressive hemicraniectomy and characterized by new onset aphasia, right facial droop, right hemiparesis, and dysarthria. She was later discharged to inpatient rehabilitation.

On follow-up, she continued to have fluctuations in memory and cognitive function, although her hemiparesis, dysarthria, aphasia, and facial droop had resolved.

The median (range) intensive care unit length of stay was 0 (0–2) days, while the median (range) hospital stay was 1 (0–6) days. The majority of patients were discharged home from the hospital (95.5%), while one patient was discharged to inpatient rehabilitation. Many patients (77.3%) in this cohort received adjuvant treatment after their LITT procedure, with 68.2% receiving chemotherapy and 50.0% receiving radiation therapy. 

Three patients (13.6%) underwent staged LITT (Cases #5, 11, and 13), and all of them tolerated the staged procedures without complication. One of these patients (Case #13) underwent a third staged treatment. Two patients underwent repeat, non-staged LITT due to residual (Case #1) or recurrent (Case #13) tumor 2 months and 2 years after initial treatment, respectively. One patient (Case #7) underwent subtotal resection due to tumor progression after LITT. Repeat pathology in this patient demonstrated malignant transformation of the tumor from grade 2 diffuse astrocytoma to grade 3 anaplastic astrocytoma.

### 3.4. Progression-Free Survival

**Figure 2:**
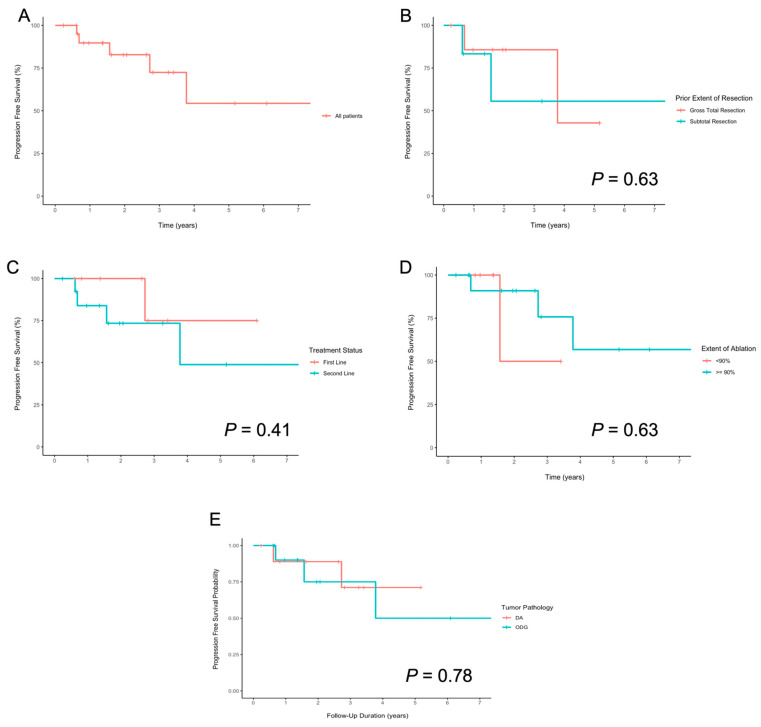
Kaplan–Meier plots showing progression-free survival (**A**) overall and stratified by (**B**) extent of prior resection, (**C**) first line versus salvage, (**D**) extent of ablation, and (**E**) tumor pathology.

Kaplan–Meier curves were used to graphically display PFS (Figure 2), and log-rank tests were used to compare the differences between groups. The cohort’s median (range) follow-up duration was 2.0 (0.2–7.5) years. In the overall cohort, the median PFS was not reached at the time of analysis; the mean (standard error) PFS was 5.2 (0.8) years. For the overall cohort, the 3-year estimated PFS was 72.5% (95% CI 47.8–97.2), and the 5-year estimate was 54.4% (18.5–90.3%). Mean, median, and estimate PFS stratified by extent of ablation, extent of resection, pathology, and treatment status can be found in Table 4. Of note, several cut-offs for extent of ablation were examined (90%, 95%, 99%), with no difference in PFS. 

Five patients (22.7%) had progression of their tumors at a median time (range) of 18.8 (7.4–45.3) months after LITT; importantly, this group was both small and heterogenous in nature. We then evaluated for factors associated with tumor progression. We considered age, extent of ablation, WHO grade, pathology, first line versus salvage, adjuvant therapy, and prior extent of resection in our analysis. We found that none of these factors were independent risk factors for PFS on univariate or multivariate analysis (Table S1), although this analysis is admittedly limited by low total number and events. 

## 4. Discussion

Presently, maximally safe surgical resection is the standard of care for *IDH1/2* mutant glioma. Although the use of LITT in the treatment of grades II and III glioma has increased over the last few decades for both primary [3,4] and recurrent gliomas [24,29], to our knowledge, this is the first multi-patient study analyzing the outcomes of *IDH1/2* mutant grade 2/3 gliomas treated with LITT. 

In our cohort of 22 patients, there was relatively equal representation of *IDH1/2* mutant oligodendroglioma and diffuse astrocytoma. LITT appeared to be tolerated well, with only three patients experiencing perioperative complications, two of which were neurologic in nature. The vast majority of patients were discharged home after a short hospital stay. These findings are in line with other studies analyzing the rate of complications after LITT for glioma, as well as other pathologies, in addition to being in line with the rate of complications seen in some craniotomy cohorts (Table 5) [30,31,32,33]. 

In our cohort, the median PFS was not reached within our median (range) follow-up time of 2.0 (0.2–7.5) years. Our 3- and 5-year PFS estimates were 72.5% and 54.4%, respectively. In our cohort, 22.7% of our patients experienced tumor progression at a median time of 18.8 months after LITT. Reported PFS after LITT in grade 2 and 3 astrocytoma and oligodendroglioma have ranged from 3 to 16 months (Table 5) [34,35,36,37]. However, data are limited because many prior case series and reports of gliomas treated with LITT did not analyze PFS duration. They instead focused on safety profile and complication rates. These cases also did not routinely report the *IDH1/2* status of the tumors, and cohorts were presumed to be a mixture of *IDH1/2* mutant and wild type. The current case series represents the first of its kind to analyze treatment outcomes in *IDH1/2* mutant glioma in particular. 

**Figure 3:**
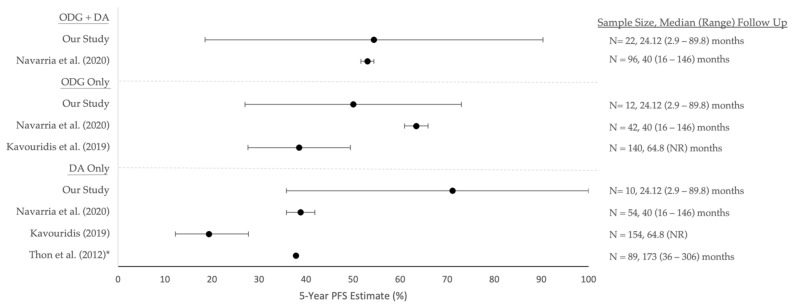
Five-year PFS estimates (95% confidence intervals) of prior surgical resection studies. Corresponding median (range) follow-up durations and sample sizes can be found on the right side for each study. * Indicates that a confidence interval was not provided or able to be calculated for that study. Abbreviations: PFS = progression-free survival, ODG = oligodendroglioma, DA = diffuse astrocytoma, NR = not reported.

As a comparison, we also looked at studies analyzing outcomes in *IDH1/2* mutant tumors treated with open resection (Table 5). In cohorts treated primarily by surgical resection and adjuvant therapy, median PFS for *IDH1/2* mutant grade 2 and 3 gliomas ranged from 46.8 to 78.0 months [18,30,38,39]. Stratified by tumor pathology, ODG median PFS ranged from 76 to 113 months [38,40], and DA from 52 to 56 months [38,40]. In addition, mean PFS and PFS estimates at various time points have been reported in the previous literature (Table 5) [31,32,39,40,41]. Our cohort did not reach the median PFS; thus, we can only conclude that our cohort’s PFS was greater than our median follow-up time of 2.0 years. The overall mean PFS, however, was 5.2 years, and our 3- and 5- year estimates of PFS were 72.5% and 54.4%, respectively. Importantly, these estimates are at least comparable those found in the surgical resection literature (Figure 3) [32,38,39,41]. 

We must acknowledge selection bias when comparing PFS in our cohort with the surgical resection literature. In many cases, LITT at our institution is offered to patients who are otherwise not suitable surgical candidates or as salvage therapy. When comparing the PFS interval between the first and second episode of recurrence in the Miller et al. cohort (PFS2, 3.1 years) with the median PFS of our patients for whom LITT was salvage or second line (3.8 years), our PFS interval was found to be similar to that found in the surgical resection cohort [18].

Several questions remain about the treatment of *IDH1/2* mutant glioma with LITT, and one is the question of malignant progression rates post-treatment. In our cohort, one patient exhibited malignant transformation approximately 7 months after LITT. Of note, this was the patient’s second overall recurrence, and the tumor molecular analysis demonstrated polysomy 7, which has been associated with malignant degeneration of low-grade glioma [33]. Overall, the proportion of patients whose tumors underwent malignant transformation was 4.5%, which is lower than reported rates in *IDH1/2* mutant glioma after resection [32]. Further validation is required to test the intriguing hypothesis that LITT may also decrease rates of malignant transformation.

This study has several limitations to acknowledge. Our cohort size, although the largest to date examining outcomes in *IDH1/2* mutant glioma after LITT, is still small, and as such is limited in its power to detect subtle-to-moderate differences between groups. For this reason, we were unable to perform subgroup analysis, particularly comparing patients treated with first-line versus salvage therapy. Additionally, we were unable to account for heterogeneity in pre-LITT and adjuvant treatments, including those who underwent surgical resection before LITT and those who did not. Our median duration of follow-up was shorter than prior literature analyzing outcomes in *IDH1/2* mutant gliomas after surgical resection, although our median approached the recommended threshold for *IDH1/2* mutant gliomas [16]. Notably, our cohort did not reach the median PFS within our duration of follow-up. While out of the scope of this preliminary, retrospective analysis, a larger, multi-institutional cohort analysis or prospective study is needed to further characterize outcomes in *IDH1/2* mutant grade 2/3 gliomas treated with LITT and further elucidate its effects on PFS compared to both open surgical resection and *IDH1/2* wild-type gliomas; this will be the focus of future work. 

## 5. Conclusions

LITT is an effective alternative to open resection in patients with glioma. In this study, we analyzed the outcomes of patients with *IDH1/2* mutant grade 2 and 3 gliomas treated with LITT at a single institution. We found that our cohort had a relatively low rate of complications that was on par with complication rates seen in craniotomy and LITT cohorts, and malignant progression after LITT was rare. The median time to progression and three- and five-year PFS estimates in our cohort were on par with those reported in the literature after surgical resection. While further, multi-institutional studies are needed to better characterize treatment outcomes after LITT in patients with *IDH1/2* mutant glioma and to elucidate risk factors for progression, the findings in this study suggest that LITT may be an effective treatment option for this molecular subtype. 

## Figures and Tables

**Table 1:** Cohort demographics and initial presentation.

Case	Age	Sex	Presenting Symptoms	Pre-LITT KPS	Pre-LITT Treatment	Lesion Location	Tumor Volume (cm^3^)
1	43.8	F	Sensory changes, headache, syncopal episodes, fatigue, change in taste/smell	90	None	L frontotemporal, insula	21.39
2	44.0	M	Seizures	80	None	R frontoparietal, cingulate gyrus	2.42
3	65.9	M	Seizures	90	None	L parietooccipital	0.84
4	35.0	F	Headache, vision problems, neurocognitive deficits, behavioral changes	70	None	R parietooccipital	10.35
5	67.3	F	Seizures, neurocognitive deficits, tremor	70	Bx	L frontotemporal, basal ganglia, insula	37.02
6	60.7	M	Seizures	80	GTR ×2, RT, C	R frontal	10.67
7	49.8	M	Seizures, speech difficulties	80	STR, RT, C	L occipital	5.95
8	46.9	F	None	100	GTR, RT,	R frontal	15.75
9	56.9	M	Muscle weakness/paralysis, speech difficulties	70	GTR ×2, RT, Bx	L frontoparietal	1.69
10	46.4	F	None	100	GTR	L frontal	1.08
11	53.3	M	None	100	STR, RT, C	R temporal, insula	22.28
12	60.0	M	Muscle weakness/paralysis, headache	80	STR, C,	R frontoparietal	10.01
13	21.8	M	Seizures	80	Bx	L frontotemporal, insula	48.38
14	47.4	F	None	100	STR, RT,	R temporal	10.29
15	36.0	M	Seizures	80	STR ×2, RT, C, GK	L temporal	3.02
16	45.4	M	None	100	GTR, RT, C, Bx	L frontal	0.38
17	31.3	M	Headache	90	None	L insula	15.91
18	48.5	M	Muscle weakness/paralysis, headache, speech difficulties	60	STR ×2, RT, C	L frontoparietal	0.18
19	44.2	F	Seizures	100	GTR, RT, C	R frontal	1.05
20	41.9	M	Earaches	90	None	L insula	3.15
21	34.3	M	None	100	GTR, RT, C	L insula	3.78, 1.46 *
22	44.8	F	Seizures	80	STR, RT, C	R temporal	5.30

Abbreviations: KPS = Karnosfsky Performance Score, F = female, M = male, L = left, R = right, GTR = gross total resection, STR = subtotal resection, RT = radiation therapy, C = chemotherapy, GK = gamma knife radiosurgery, Bx = biopsy. * This patient presented with two lesions.

**Table 2:** Histopathologic findings.

Case	Tumor Type	WHO Grade	Mutations	Ki-67 Index	Tumor Mutational Burden
1	Diffuse astrocytoma	2	*IDH1*, *TP53*, *ATRX*	≈4	1
2	Oligodendroglioma	2	*IDH2*, *TERTp*, *CIC*, 1p/19q co-deletion	17	0
3	Oligodendroglioma	2	*IDH1*, *TERTp*, *CIC*, 1p/19q co-deletion	6	3
4	Diffuse astrocytoma	2	*IDH1*, *TP53*, *ATRX*	2.5	6
5	Oligodendroglioma	2	*IDH1*, 1p/19q co-deletion	NT	NT
6	Diffuse astrocytoma	2	*IDH1*, *TP53*, *ATRX*	<10	NT
7	Diffuse astrocytoma	2	*IDH1*	5.7	NT
8	Diffuse astrocytoma	2	*IDH1*, *TP53*, *ATRX*	2.3	3
9	Oligodendroglioma	2	*IDH1*, 1p/19q co-deletion	8.2	NT
10	Oligodendroglioma	2	*IDH1*, 1p/19q co-deletion	2.1	NT
11	Oligodendroglioma	2	*IDH1*, *TERTp*, *CIC*, 1p/19q co-deletion	3.7	0
12	Oligodendroglioma	2	*IDH1*, *TERTp*, *CIC*, 1p/19q co-deletion	NT	42
13	Diffuse astrocytoma	2	*IDH1*	<5	NT
14	Diffuse astrocytoma	3	*IDH1*, *TP53*, *ATRX*	1.4	1
15	Oligodendroglioma	3	*IDH1*, *TERTp*, *CIC*, 1p/19q co-deletion	40	16
16	Oligodendroglioma	3	*IDH1*, *TERTp*, 1p/19q co-deletion	9.9	13
17	Diffuse astrocytoma	3	*IDH1*, *TP53*, *ATRX*	NT	NT
18	Oligodendroglioma	3	*IDH1*, 1p/19q co-deletion	24	NT
19	Oligodendroglioma	3	*IDH1*, *TERTp*, *CIC*, 1p/19q co-deletion	20	NT
20	Diffuse astrocytoma	3	*IDH1*, *TP53*, *ATRX*	5–10	4
21	Diffuse astrocytoma	3	*IDH1*, *TP53*, *ATRX*	31.5	1
22	Oligodendroglioma	3	*IDH1*, *TERTp*, 1p/19q co-deletion	50	3

Abbreviations: NT = not tested.

**Table 3:** Operative details and outcomes.

Case	First Line vs. Salvage	Indication for LITT	EOA (%)	Perioperative Complications	Adjuvant Tx	Repeat LITT	Time to Progression (Months)	Follow-Up (Months)
1	First line	Tumor location	80	Severe edema, new FND	RT, C	Yes	No progression	9.85
2	First line	Tumor location	95	None	RT, C	No	No progression	7.36
3	First line	Unknown	85	None	RT, C	No	No progression	16.52
4	First line	Tumor location	95	None	RT, C	No	No progression	33.80
5	First line	Shorter LOS	65	None	RT, C	No	No progression	73.13
6	Salvage	Refractory to Tx	95	None	None	No	No progression	62.12
7	Salvage	Recurrence	NR	None	STR, RT, C	No	7.42	70.14
8	Salvage	Recurrence	95	None	RT, C	No	No progression	19.48
9	Salvage	Recurrence	98	None	C	No	45.34	88.50
10	Salvage	Recurrence	100	None	None	No	No progression	23.55
11	Salvage	Recurrence	80	None	C	No	18.82	18.82
12	Salvage	Recurrence	80	Seizure	None	No	No progression	16.29
13	First line	Aborted craniotomy	70	None	None	Yes	32.69	85.32
14	Salvage	Recurrence	NR	None	RT, C	No	No progression	39.13
15	Salvage	Recurrence	99	None	RT, C	No	8.25	15.01
16	Salvage	Recurrence	80	None	C, GK	No	No progression	11.66
17	First line	Lower percieved risk	95	None	C	No	No progression	31.57
18	Salvage	Recurrence	100	None	RT	No	No progression	89.82
19	Salvage	Recurrence	100	DVT	None	No	No progression	24.77
20	First line	Favorable safety profile	80	None	None	No	No progression	40.97
21	Salvage	Recurrence	99	None	RT, C	No	No progression	2.89
22	Salvage	Recurrence	95	None	C x2	No	No progression	7.78

Abbreviations: EOA = extent of ablation, FND = focal neurologic deficit, LOS = length of stay, Tx = treatment, DVT = deep vein thrombosis, RT = radiation therapy, C = chemotherapy, GK = gamma knife, STR = subtotal resection, NR = not reported.

**Table 4:** PFS stratified by extent of resection, treatment status, extent of ablation, and pathology.

Variable	Mean (SE) PFS, Years	Median (SE) PFS, Years	Three-Year PFS (SE) Estimate	Five-year PFS (SE) Estimate
Prior extent of resection				
Gross total resection	3.9 (0.7)	3.8 (NC)	85.7% (13.2%)	42.9% (31.0%)
Subtotal resection	4.7 (1.5)	Not reached	55.6% (24.8%)	55.6% (24.8%)
Treatment status				
First line	5.3 (0.7)	Not reached	75.0% (21.7%)	75.0% (21.7%)
Salvage	4.9 (1.0)	3.8 (NC)	73.4% (13.4%)	49.0% (21.9%)
Extent of ablation				
<90%	2.5 (0.7)	1.6 (NC)	50.0% (35.4%)	50.0% (35.4%)
≥90%	5.4 (0.9)	Not reached	75.8% (15.6%)	56.8% (20.1%)
Pathology				
DA	4.2 (0.6)	Not reached	71.1% (18.0%)	71.1% (18.0%)
ODG	5.0 (1.1)	3.8 (NC)	75.0% (15.8%)	50.0% (23.0%)

Abbreviations: SE = standard error, NC = not calculated, DA = diffuse astrocytoma, ODG = oligodendroglioma.

**Table 5:** Studies analyzing PFS in *IDH1/2* mutant cohorts.

Study (Year)	WHO Grade	Median (Range) Follow-Up, Months	Pathology	Median (95% CI) PFS, months	Three-Year PFS Estimate (95% CI)	Five-Year PFS Estimate (95% CI)	Ten-Year PFS Estimate (95% CI)	Complication Rates ^a^	Notes
Our study	II, III	24.1 (2.9–89.8)	ODG + DA (*n* = 22)	Median not reached	72.5% (57.8–97.2)	54.4% (18.5–90.3)	NR	Perioperative: 14%	Mean (SE) PFS: ODG + DA: 62.4 (10.0) ODG: 59.9 (13.2) DA: 50.76 (6.96)
			ODG (*n* = 12)	45.6	75.0% (44.1–100)	50.0% (27.0–73.0)	NR		
			DA (*n* = 10)	Median not reached	71.1% (35.8–100)	71.1% (35.8–100)	NR		
		**Craniotomy Cohorts**							
Navarria et al. (2020)	III	40 (16–146)	ODG + DA (*n* = 96)	69 (51–89)	62.4% (61.3–63.5)	53.0% (51.6–54.4)	NR	Perioperative: 16% Worsening of preoperative deficits: 14%	
			ODG (*n* = 42)	76 (32–89)	63.4% (60.9–65.9)	63.4% (60.9–65.9)	NR		
			DA (*n* = 54)	52 (34–57)	59.9% (57.8–62.0)	38.8% (35.8–41.8)	NR		
Patel et al. (2018)	II	44.4 (0.6–187.2)	ODG + DA (*n* = 52)	78 (NR)	88.8% (79.6–98.1)			NR	Malignant PFS only
Kavouridis et al. (2019)	II	64.8 (NR)	ODG (*n* = 140)	NR	NR	38.5% (27.6–49.4)	24.1% (12.8–37.4)	NR	
			DA (*n* = 154)	NR	NR	19.3% (12.2–27.7)	3.2% (0.6–9.6)		
Tom et al. (2019)	II	NR	ODG (*n* = 18)	113 (NR)	NR	NR		NR	All patients with GTR
			DA (*n* = 30)	56 (NR)	NR	NR			
Choi et al. (2020)	II	66.9 (5.3–171.3)	ODG (*n* = 45)	NR	NR	NR	73.6% (NR)	NR	
			DA (*n* = 80)	NR	NR	NR	32.5% (NR)		
Pal’a et al. (2019)	II	72 (95% CI 57.6–75.6)	ODG + DA (*n* = 144)	46.8 (NR)	NR	NR	NR	NR	
Miller et al. (2019)	II, III	76.8 (NR)	ODG + DA (*n* = 275)	PFS1: 68.4 (56.4–76.8) PFS2: 37.2 (25.2–49.2)	NR	NR	NR	NR	PFS1: resection to first recurrence PFS2: first recurrence to second recurrence
			DA (*n* = 180)	68.2 (NR)	NR	NR	NR		
			ODG (*n* = 95)	67.9 (NR)	NR	NR	NR		
Thon et al. (2012)	II	173 (36–306)	DA (*n* = 89)	47 (range 35–60)	NR	37.8% (NR)	10.5% (NR)	NR	Supratentorial only
		**LITT Cohorts**							
Mohommadi et al. (2014) *	III	7.2 (0.1–23.0)	DA (*n* = 6) ODG (*n* = 4)	5.6 (NR)	NR	NR	NR	Any complication: 37% Worsening of preoperative deficits: 20% Seizure: 3% Infection: 6%	
Leonardi and Lumeta (2002) *	II, III	NR	Low-grade DA (*n* = 7)	Mean: 16 (9–233)	NR	NR	NR	Neurologic deterioration: 17% Seizure: 4% Infection: 8%	
			Anaplastic ODG + DA (*n* = 11)	Mean: 10 (6–14)	NR	NR	NR		
Reimer et al. (1998) *	III	12 (NR)	DA (*n* = 3)	6 (range 6–12)	NR	NR	NR	Transient aphasia: 25%	
Murayi et al. (2020) *	III	NR	DA (*n* = 2)	Pt 1: 2.9 Pt 2: death POD3	NR	NR	NR	Permanent morbidity: 46% Perioperative mortality: 15%	

Abbreviations: PFS = progression-free survival, CI = confidence interval, ODG = oligodendroglioma, DA = diffuse astrocytoma, SE = standard error, NR = not reported, GTR = gross total resection. * Indicates cohorts with unclear IDH mutational status. ^a^ Complication rates were taken as a proportion of the total cohort, including those that were not grades 2 and 3.

## Data Availability

The dataset used and/or analyzed during the current study are available from the corresponding author on reasonable request.

## Supplementary Materials

The following are available online at https://www.mdpi.com/article/10.3390/curroncol29040209/s1, Table S1: Univariate time to event and regression analysis.
