# Supplementary material for: Laser Interstitial Thermal Therapy in Grade 2/3 IDH1/2 Mutant Gliomas: A Preliminary Report and Literature Review
Source: Curr Oncol. 2022 Apr 8;29(4):2550–63. doi: 10.3390/curroncol29040209 (PMC9028957; doi:10.3390/curroncol29040209)
Supplement: Supplementary file 1 [file curroncol-29-00209-s001.zip › curroncol-1576477-supplementary.pdf]

Laser Interstitial Thermal Therapy in Grade 2/3 *IDH1/2* Mutant Gliomas: A Preliminary Report and Literature Review

Gabrielle W. Johnson [1], Rowland H. Han [1], Matthew D. Smyth [2], Eric C. Leuthardt [1,3] and Albert H. Kim [1,3,*]

Table S1. Univariate Time to Event and Regression Analysis.

Variable	Progression	No Progression	Univariate Time to Event Analysis[a]	Univariate Regression Analysis
	N = 5	N = 17	P-value	P-value
Age				
< 40 years	2 (40.)	3 (17.6)	Ref	Ref
≥ 40 years	3 (60.0)	14 (82.4)	0.189	0.308
WHO Grade				
Grade II	4 (80.0)	9 (52.9)	Ref	Ref
Grade III	1 (20.0)	8 (47.1)	0.454	0.298
Pathology				
DA	2 (40.0)	8 (47.1)	Ref	Ref
ODG	3 (60.0)	9 (52.9)	0.775	0.781
Treatment Indication				
1st Line	1 (20.0)	7 (41.2)	Ref	Ref
Salvage	4 (80.0)	10 (58.8)	0.413	0.399
Prior EOR	N = 4	N = 10		
GTR	2 (50.0)	6 (60.0)	Ref	Ref
STR	2 (50.0)	4 (40.0)	0.630	0.733
Extent of Ablation				
< 90%	1 (25.0)	5 (31.3)	Ref	Ref
≥ 90%	3 (75.0)	11 (68.8)	0.630	0.808
Post-LITT Adjuvant Tx			0.167	
Chemotherapy Only	2 (40.0)	3 (25.0)	NC	Ref
RT + Chemotherapy	0 (0)	8 (66.7)	NC	>0.999
RT Only	0 (0)	1 (8.3)	NC	0.999
Other	2 (40.0)	0 (0)	Not Included	Not Included
None	1 (20.0)	0 (0)	Not Included	Not Included

[a] Calculated using the Mantel-Cox log-rank test; Abbreviations: DA = diffuse astrocytoma, ODG = oligodendroglioma, EOR = extent of resection, GTR = gross total resection, STR = subtotal resection, Tx = treatment, RT = radiation therapy, NC = not calculated.
